# Imlifidase for Kidney Transplantation of Highly Sensitized Patients With a Positive Crossmatch: The French Consensus Guidelines

**DOI:** 10.3389/ti.2023.11244

**Published:** 2023-06-28

**Authors:** Lionel Couzi, Paolo Malvezzi, Lucile Amrouche, Dany Anglicheau, Gilles Blancho, Sophie Caillard, Marine Freist, Gwenda Line Guidicelli, Nassim Kamar, Carmen Lefaucheur, Christophe Mariat, Alice Koenig, Johan Noble, Olivier Thaunat, Antoine Thierry, Jean-Luc Taupin, Dominique Bertrand

**Affiliations:** ^1^ Centre Hospitalier Universitaire de Bordeaux, Bordeaux, France; ^2^ CNRS-UMR 5164 Immuno ConcEpT, Université de Bordeaux, Bordeaux, France; ^3^ Centre Hospitalier Universitaire de Grenoble, La Tronche, France; ^4^ Hôpital Necker-Enfants Malades, Paris, France; ^5^ Centre Hospitalier Universitaire (CHU) de Nantes, Nantes, France; ^6^ Hôpital Civil, Strasbourg, Strasbourg, France; ^7^ Centre Hospitalier Emile Roux, Le Puy-en-Velay, France; ^8^ Hôpital des Enfants, Centre Hospitalier Universitaire de Bordeaux, Bordeaux, France; ^9^ Centre Hospitalier Universitaire de Toulouse, Toulouse, France; ^10^ Hôpital Saint-Louis, Paris, France; ^11^ Centre Hospitalier Universitaire (CHU) de Saint-Étienne, Saint-Etienne, France; ^12^ Hospices Civils de Lyon, Lyon, France; ^13^ Centre Hospitalier Universitaire (CHU) de Poitiers, Poitiers, France; ^14^ Centre Hospitalier Universitaire (CHU) de Rouen, Rouen, France

**Keywords:** kidney transplantation, desensitization, imlifidase, highly sensitized patients, positive crossmatch

## Abstract

Imlifidase recently received early access authorization for highly sensitized adult kidney transplant candidates with a positive crossmatch against an ABO-compatible deceased donor. These French consensus guidelines have been generated by an expert working group, in order to homogenize patient selection, associated treatments and follow-up. This initiative is part of an international effort to analyze properly the benefits and tolerance of this new costly treatment in real-life. Eligible patients must meet the following screening criteria: cPRA ≥ 98%, ≤ 65-year of age, ≥ 3 years on the waiting list, and a low risk of biopsy-related complications. The final decision to use Imlifidase will be based on the two following criteria. First, the results of a virtual crossmatch on recent serum, which shall show a MFI for the immunodominant donor-specific antibodies (DSA) > 6,000 but the value of which does not exceed 5,000 after 1:10 dilution. Second, the post-Imlifidase complement-dependent cytotoxicity crossmatch must be negative. Patients treated with Imlifidase will receive an immunosuppressive regimen based on steroids, rATG, high dose IVIg, rituximab, tacrolimus and mycophenolic acid. Frequent post-transplant testing for DSA and systematic surveillance kidney biopsies are highly recommended to monitor post-transplant DSA rebound and subclinical rejection.

## Background on Imlifidase

Imlifidase is a recombinant cysteine protease derived from *Streptococcus pyogenes* and produced in *Escherichia coli*, which has the ability to cleave and degrade all human IgGs [1]. Four to 6 hours after Imlifidase infusion, the entire IgG pool is degraded into F(ab’)2 and Fc fragments [2]. *In vitro*, Imlifidase inhibits HLA antibody-mediated NK cell activation and antibody-dependent cell-mediated cytotoxicity [3]. Imlifidase degrades also the IgG of the B cell Receptor (BCR), inhibiting BCR-mediated cell signal, transiently preventing memory B cell response to antigenic stimulation and their transition into antibody-producing cells [4].

Two clinical studies have been designed to determine whether Imlifidase could inactivate IgG donor-specific antibodies as a desensitization strategy in highly sensitized candidates for kidney transplantation. In the phase I/II study, 25 patients were transplanted in Sweden and United States. Among them, 18 had a positive flow cytometry crossmatch (FCXM) and 2 a positive complement-dependent cytotoxicity crossmatch (CDCXM) [2]. In the phase II study (Highdes Trial), 19 patients with an incompatible living or deceased donor from the United States, Sweden, and France were included. Among them, 7, 18, 2, and 8 had respectively a positive T-cell FCXM, positive B-cell FCXM, positive T-cell CDCXM, and positive B-cell CDCCXM. The primary efficacy endpoint was the ability of Imlifidase to convert a positive XM to a negative one. Conversion of baseline positive XM to negative within 24 h after Imlifidase treatment occurred in 89.5% (*n* = 17) of the 19 patients [5]. In the follow-up study including all the patients transplanted after Imlifidase desensitization, the antibody-mediated rejection rate (AMR) was at 39%. Three-year death-censored graft survival was 93% in patients with AMR and 77% in the others. Three-year patient survival was 85% in patients with AMR and 94% in the others [6]. No safety signal was reported.

Based on these data, Imlifidase is now indicated as a desensitization agent of highly sensitized adult kidney transplant patients with positive crossmatch against an available ABO-compatible deceased donor. Imlifidase received a conditional marketing authorization valid throughout the European Union on 25 August 2020 (https://www.ema.europa.eu). On 23 February 2022, the French health agency authorized an early access to Imlifidase (Idefirix). On 16 August 2022, a panel of 12 transplant nephrologists and four immunologists (including two HLA experts) was convened by The French Society of Transplantation (SFT), the French-speaking Society of Nephrology, Dialysis and Transplantation (SFNDT) and the French Society of Histocompatibility and Immunogenetics (SFHI) to propose recommendations for patient selection, choice of antibodies characteristics, treatment and follow-up in order to homogenize practices. The expert panel used the Grading of Recommendations Assessment, Development and Evaluation system for a systematic weighting of the strength of the recommendation (high: A, moderate: B, low: C, very low: D) and quality of evidence (strong: 1, weak: 2) [7]. Finally, the guidelines were discussed and approved with the French agency in charge of organ regulation (Agence Nationale de la Biomedecine). The objective of these recommendations is to propose a common framework for teams using Imlifidase in order to analyze properly the benefits and tolerance of this new treatment in real-life.

## Available Strategies in Highly Sensitized Patients: The Place of Imlifidase

Very recently, the ENGAGE working group (EuropeaN Guidelines for the mAnagement of Graft rEcipients) from ESOT proposed an updated definition of sensitization, stratifying the humoral risk of candidates for solid organ transplantation [8]. Among patients with day 0 donor-specific antibody (DSA), the risk of AMR is the highest in positive CDCXM patients, a situation which requires a desensitization protocol to avoid hyperacute rejection (ENGAGE category 1). Positive FCXM patients have a lower risk of AMR but these patients also require an increased immunosuppression (ENGAGE category 2). Patients with day 0 DSA but a negative crossmatch are also at increased risk of AMR but have an acceptable medium-term graft survival (ENGAGE category 3). This stratification is supported by the studies published by Orandi et al. which showed that graft survival, patient survival and risk of AMR were highly associated with the positivity of the FCXM and the CDCXM [9, 10]. Patients with a positive FCXM have a 35% risk of AMR, which increases to 50% in those with a positive CDCXM [11]. Five-year graft loss is also poor at 30% in positive FCXM recipients and 40% in positive CDCXM [10].

The use of Imlifidase should be reserved for patients unlikely to be transplanted under the available kidney allocation system including the prioritization program for highly sensitized patients (https://www.ema.europa.eu). The French kidney allocation system (KAS) has changed in 2015 and introduced a unified allocation score to be applied locally for one kidney and nationally for the other. In our KAS, highly sensitized patients have access to a national priority program. A recent paper published recently summarizes all these rules [12]. In France, the degree of sensitization (cPRA) reflects the percentage of incompatible donors with HLA antigens against which the patient has preformed anti-HLA antibodies, among all isogroup donors collected on the national territory, during the past 5 years. Highly sensitized patients are defined by a recent cPRA ≥ 70% and a peak cPRA ≥ 85%. In a recent review, Mamode et al. summarized all the available options for transplanting highly sensitized transplant candidates [13].

**FIGURE 1:**
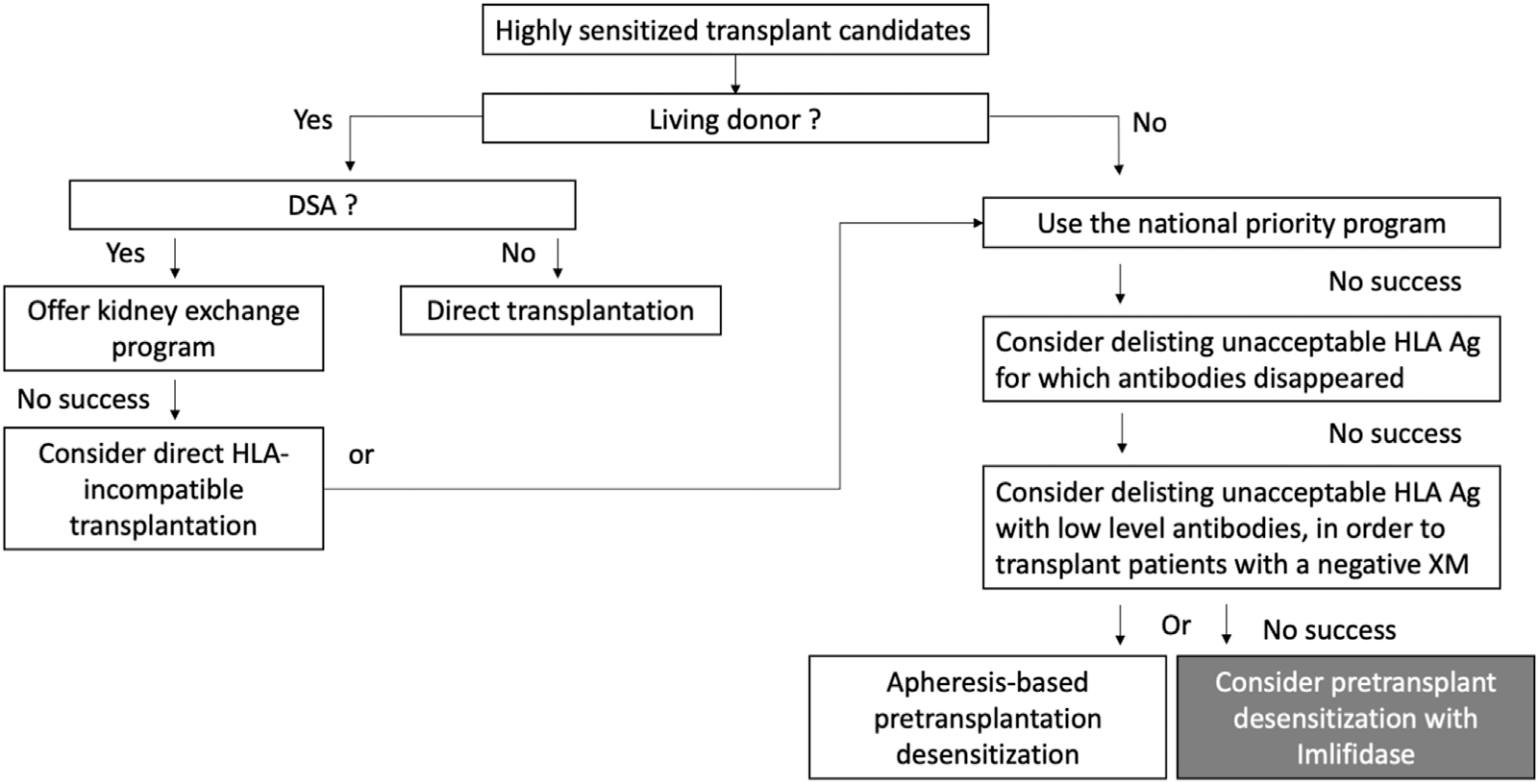
The place of Imlifidase among the available strategies for highly sensitized patient transplantation. Adapted from Mamode et al. [13].

A living-donor transplantation must be considered for all these patients and three strategies are available: a direct transplantation with an HLA-compatible donor, an indirect transplantation thanks to a kidney exchange program, and finally a direct HLA-incompatible transplantation (Figure 1). Although patients transplanted with preformed DSA have globally a greater risk for AMR, this humoral risk greatly varies and can be stratified according to the results of the crossmatch assays, as proposed in the ENGAGE classification [8].

Living-donor transplantation options are often limited, and most highly sensitized patients are transplanted with a deceased donor. In the United States, 73% of transplantations are performed with a deceased donor in patients with a cPRA < 80%. This rate reaches 95%–98% in patients with a cPRA>98% [14]. If they are not transplanted with a compatible donor, transplant teams have the possibility to consider delisting unacceptable HLA antigens for which antibodies disappeared. They also have the possibility to consider delisting unacceptable HLA antigens with low level HLA antibodies (Figure 1). The objective of this last strategy is to perform DSA positive but negative XM transplantations (i.e., ENGAGE category 3) [15–17]. But these strategies are very rarely applicable to highly sensitized candidates with persistent high-level HLA antibodies for whom positive XMs are expected (ENGAGE categories 1 and 2). For these patients, many pretransplant desensitization strategies have been tested in order to lower the titer of preformed DSA. These strategies were initially based on IVIg [18–20], then rituximab and IVIg [21], and more recently Bortezomib and apheresis [22], but their efficacy is still discussed. Sequential or single pre-transplant apheresis-based desensitization programs have also been developed by a few transplant teams [23–25]. For instance, the Vienna group proposed to 27 deceased-donor kidney transplant recipients a pre-transplant immunoadsorption for obtaining a negative CDCXM, but the rate of AMR was high (41%) [26]. Faced with the complexity of some of these strategies, complement inhibitors were also tested in a prospective trial with unconvincing results [27].

In France, Imlifidase is now indicated for replacing these strategies in the desensitization treatment of these patients who have a positive crossmatch against an HLA-incompatible deceased donor (Figure 1). Although additional data on long-term graft function and survival are required in patients treated by Imlifidase, the European Medicine Agency has decided that this new treatment addressed an unmet medical need (https://www.ema.europa.eu).

## Patient Selection Criteria

### Patients Eligible for This Treatment

#### Recipient With cPRA ≥ 98% (Calculated on the Last Serum)

**FIGURE 2:**
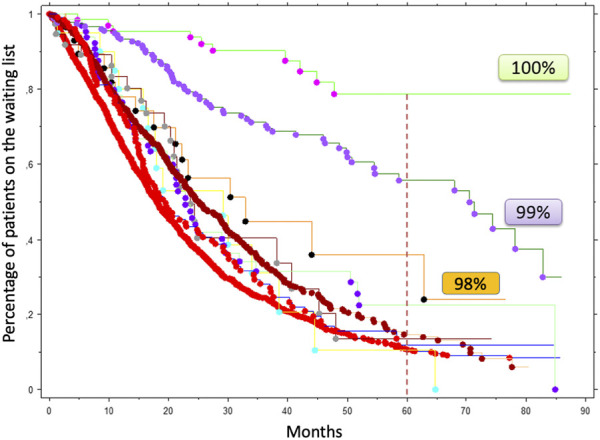
Access to a kidney transplantation according to cPRA in a French region including more 3,096 candidates listed between July 2009 and December 2015. Patients with a cPRA > 85% were included in the national priority program. These data were kindly given by D. Bertrand and collaborators who conducted this retrospective analysis in four transplant centers (Amiens, Caen, Lille, Rouen).

Given the expected high rate of AMR, the use of Imlifidase should be reserved for patients unlikely to be transplanted. Importantly, not all highly sensitized patients have the same access to a transplant. In a French region with more than 3,000 candidates awaiting a kidney transplantation, it was observed that patients with cPRA ≥ 98% had more difficult access to a compatible donor even though they were included in the national priority program (Figure 2). Based on these data, we chose a threshold of cPRA ≥ 98% (calculated on the last serum) to authorize a patient to receive Imlifidase in France (IC). However, it is important to note that the French cPRA is not comparable with cPRA used in other countries. For instance, in Australia, access to transplantation is poor for those with a cPRA of 95%–98% and even worse for those with cPRA ≥ 99% [28]. In the United States, access to transplantation becomes very limited for patients with a cPRA ≥ 99% [14]. Based on that observation, the FDA considers that only patients with cPRA ≥ 99.9 should be targeted to desensitization.

#### Recipient Age ≤ 65 years

Orandi et al. showed that positive crossmatch patients had a significantly higher risk of death than compatible patients [10]. In recipients older than 70 years, the two main causes of death are infection and cardiovascular diseases [29]. In line with these observations, patients undergoing HLA desensitization before kidney transplantation are particularly exposed to infectious diseases and cardiovascular events [23], and Avery et al. reported that the risk of infectious disease increased with the intensity of desensitization before kidney transplantation [30]. Based on these data, we propose that recipient age should not exceed 65 years (1D).

#### Time on the Waiting List ≥ 3 years

The French acceptable mismatch program improved access to transplantation for highly sensitized patients with a low risk of AMR, as described in the Eurotransplant program [31]. In order to maintain some equity of access to transplantation for all candidates, we propose that the patient wait for at least 3 years on the waiting list before being offered a transplant with an Imlifidase-based desensitization (2D). It is important to note that this period of time was chosen arbitrarily based on the median time on the waiting list in France which is currently at 2 years (www.agence-biomedecine.fr).

#### Number of Previous Kidney Transplantations From 0 to 2 (Multidisciplinary Consensus Required If > 2 Previous Transplantations)

In order to maintain some equity of access to transplantation for all candidates, and to minimize the surgical risk, we do not recommend to perform kidney transplantation with Imlifidase in patients with a history of more than two kidney transplantations (2D).

#### Transplant Biopsy With a Low Risk of Complication

As the probability to develop an AMR and therefore to undergo a transplant biopsy is very high in Imlifidase-based desensitized recipients, we recommend to select patients with an anticipated low risk of biopsy-related complications (1D).

#### Patient Information

Patients should be informed of the implications of desensitization, how it is performed, the expected benefits and risks involved (1A).

### Transplant Unit Profile

In the early post-transplantation period, AMR occurs frequently following Imlifidase desensitization. In this situation, prompt plasmapheresis sessions are highly recommended [32]. Therefore, centers must be equipped to perform round the clock apheresis treatment in the case of AMR (1A).

### Donor Profile

Given the expected high rate of AMR in patients desensitized with Imlifidase, it is important to avoid a delayed graft function secondary to poor quality of the donated kidney which could interfere in the management of an early AMR. Donor characteristics associated with a high risk of delayed graft function are old age, extended criteria donor, donation after cardiac death, warm ischemia, long ischemia time, and severe acute kidney injury. According to Aubert et al. preformed DSA and cold ischemia time are the two main independent determinants of outcome of expanded criteria donor (ECD) transplantation. Recipients of ECD kidneys with circulating DSA showed a 5.6-fold increased risk of graft loss compared with all other transplant therapies (*p* < 0.001) [33]. In this context we recommend that older donors, donation after cardiac death, long ischemia time, and acute kidney injury should be avoided as much as possible (1C).

**FIGURE 3:**
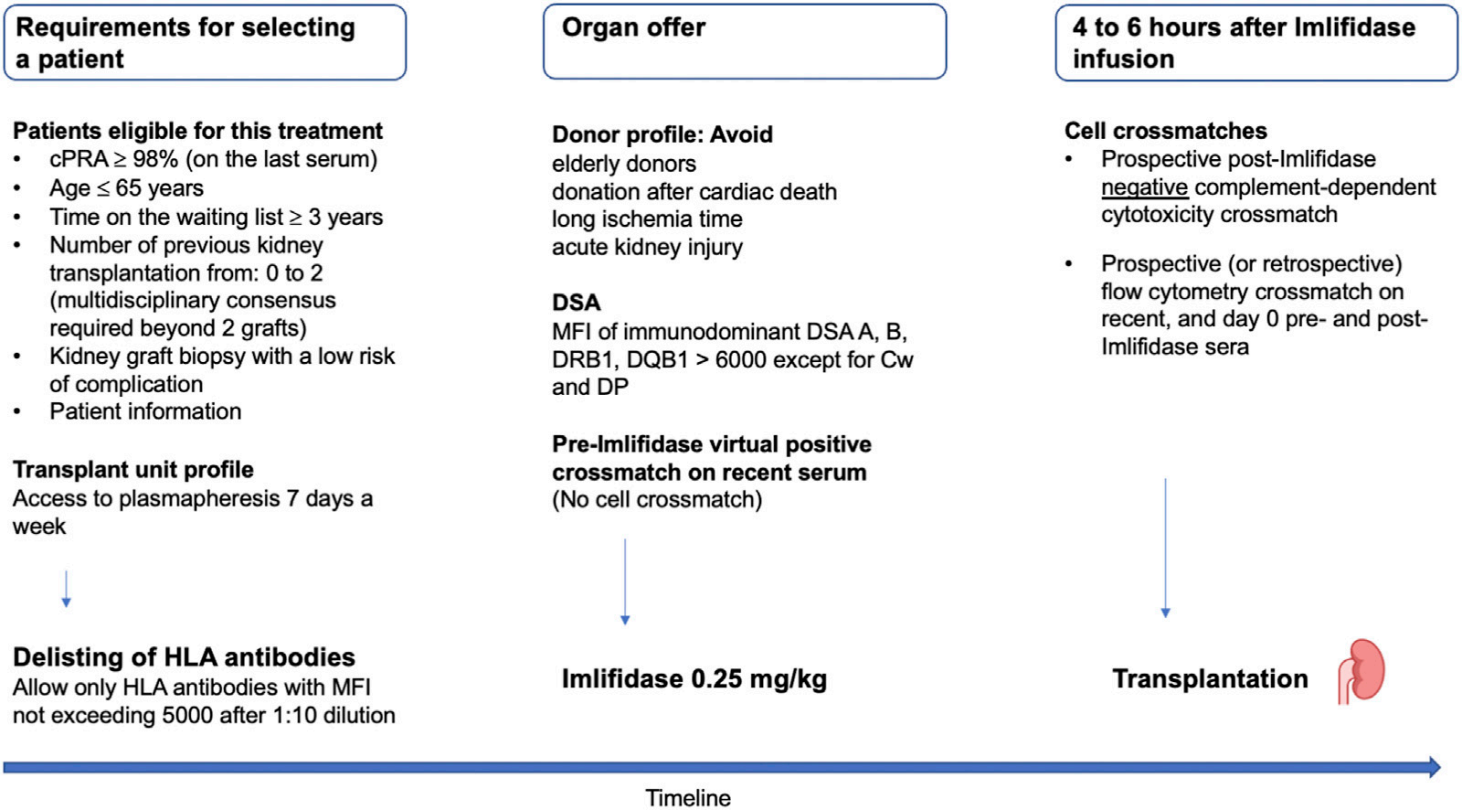
French criteria for selecting highly sensitized patients eligible to Imlifidase, permitted DSA and the timeline of crossmatches. cPRA, calculated panel reactive antibody; DSA, donor-specific antibody; MFI, mean fluorescence intensity.

These recommendations are summarized in Figure 3.

## DSA Characteristics and Crossmatches

### Delisting of HLA Antibodies With a Mean Fluorescence Intensity <5,000 After 1:10 Dilution

After kidney transplantation with Imlifidase, rebound of DSA occurs frequently with an increased risk of AMR [5]. Currently, we do not have a tool able to predict this post-transplant DSA rebound. In the pooled Imlifidase 3-year follow-up analysis, the only variable associated with AMR was the pre-Imlifidase mean fluorescence intensity (MFI) level [6]. However, the Single Antigen Flow Bead (SAFB) assay displays a progressive saturation effect of the measured MFI when the antibody load increases, leading to its underestimation. Serial sera dilutions are reported to be helpful to estimate true alloantibody levels (cPRA) in highly sensitized kidney allograft candidates [34] and to evaluate DSA strength [35]. Moreover, measurement of pre-transplant serum dilutions can be used to determine unacceptable antigens, as well as the likelihood for successful HLA antibody reduction with desensitization [36]. Serum dilution and titration studies can help determining whether desensitization is likely to be successful in removing enough HLA antibody to avoid hyperacute rejection and plan the desensitization strategy. For instance, Pinelli et al. showed that transplant candidates with DSAs of titer ≥1:1,024 at baseline were unlikely to achieve sufficient DSA reduction with PP/IVIg alone [37].

Our objectives were to limit the risk of rebound and more importantly to accept DSA that could be removed efficiently by apheresis sessions in case of rebound. Therefore, we recommend to only delist, those with a SAFB MFI below 5,000 after a 1:10 dilution (One Lambda assay) (2D). This recommendation increases significantly the cost of HLA testing and requires at the time of patient selection a delisting of all HLA antigens against which the MFI of the preformed HLA antibodies are < 5,000 after a 1:10 dilution. We recommend to update the delisted HLA antigens every 3 months until transplant offer. All preformed DSA must be still below MFI 5000 on the last diluted serum at the time of transplant offer.

### An MFI of Pre-Imlifidase Immunodominant DSA A, B, DRB1, DQB1 > 6,000 (LSAB One Lambda)

Based on the ENGAGE recommendations, our goal was to propose Imlifidase to patients with a positive pre-Imlifidase FCXM (category 2) or CDCXM (category 1). However, we chose to offer Imlifidase based on a virtual XM and not a cellular XM, in order to reduce the ischemia time. To circumvent this problem, we chose to use an MFI threshold capable of predicting the positivity of a FCXM or a CDCXM.

Vo et al. reported the rate of AMR in 226 highly sensitized patients who received transplants after desensitization, and concluded that the DSA-relative intensity scores at transplant was a strong predictor of AMR [38]. By using the assay from the One Lambda company on 432 sera also tested in T-cell XMs, Visentin et al. showed that the SAFB MFI threshold predicting a T-cell FCXM positivity was comprised between 4,400 and 6,200 for class I DSA [39]. The threshold predicting a T-cell CDCXM positivity was comprised between 8,900 and 13,600. To date, data from the other SAFB assay, from the Immucor company, are lacking in the literature. Furthermore, it has been largely demonstrated that circulating complement-activating anti-HLA DSAs had a significant deleterious impact on solid organ transplant survival and risk of rejection [40]. The C1q and C3d assay results can be efficiently predicted by the IgG SAFB MFI once complement interference is annihilated [41, 42]. For instance, Courant et al. showed that an MFI > 3,844 predicted C1q assay positivity with 87.0% sensitivity and 93.5% specificity [42].

Based on these data, we chose to offer Imlifidase only if the SAFB MFI of the immunodominant DSA (One Lambda assay) on a recent serum (less than 3 months) is above 6,000 at the time of the transplant offer (2C). We suggest that transplantations can be performed without Imlifidase if the MFI of the immunodominant DSA is less than 6,000. Other treatment options can be discussed in these situations, such as plasmapheresis and IVIg [43, 44]. A limit of this approach is the high inter-laboratory variability of MFI values.

Only DSA against A, B, DRB1, DQB1 HLA molecules were considered. It has been reported that Cw and DP DSA were associated with AMR and graft loss [45]. However, not all Cw and DP antibodies are pathogenic. For instance, 31.6% of Cw DSA are anti-denatured HLA antibodies associated with negative crossmatch and excellent graft outcome [46]. For these reasons, we did not consider DSA against Cw and DP.

### A Pre-Imlifidase Virtual Positive Crossmatch on a Recent Serum Predicting a Positive Cell-Based Crossmatch

We do not recommend to perform a cell-based crossmatch before Imlifidase infusion in order to reduce the total ischemia time (1D). At the time of organ offer, the recipient must have at least one DSA A, B, DRB1, DQB1 with a MFI > 6,000 among all the preformed HLA antibodies which were delisted (because of a MFI < 5,000 after a 1:10 dilution).

### A Post-Imlifidase Negative CDCXM (Performed Between 4 and 6 h After Imlifidase Infusion)

A post-Imlifidase negative CDCXM is mandatory to authorize kidney transplantation. CDCXM must be performed prospectively by integrating relevant historical sera and day-zero sera, including pre- and post-Imlifidase sera (4–6 h) (1A). If the CDCXM is positive, we recommend not infusing a second dose of Imlifidase and rejecting the transplant offer.

### A Prospective or Retrospective FCXM on Recent, and Day 0 Pre- and Post-Imlifidase Serum Must Be Performed

The FCXM result has no impact on the decision of transplantation. A transplantation can be performed with a positive FCXM as long as the CDCXM is negative. A FCXM is mandatory for stratifying the humoral risk of candidates receiving Imlifidase (1C) [8].

These recommendations are summarized in Figure 3. Importantly, we recommend that both CDC and FCXM crossmatches are performed with an anti-Rituximab mouse monoclonal antibody (10C5 clone, ABNOVA^®^) if the patient has received Rituximab before transplantation (see next chapter) [47, 48].

## Associated Therapies

**FIGURE 4:**
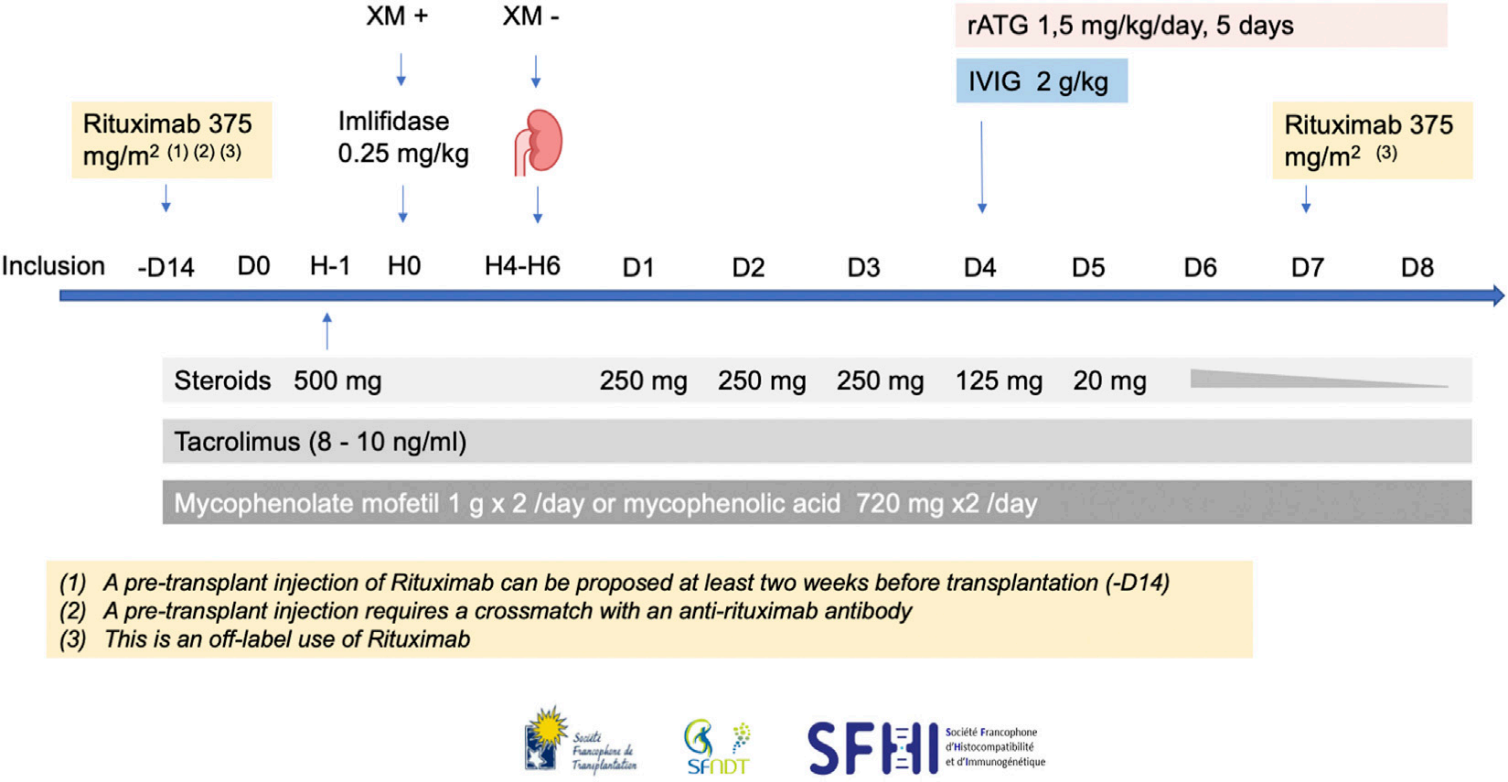
The French consensus guidelines for therapies associated with Imlifidase in highly sensitized patients transplanted with a positive crossmatch. H, hour; rATG, rabbit antithymocyte globulin; IVIg, intravenous immunoglobulin.

Imlifidase must be given as a single dose (0.25 mg/kg, IV in 15 min) prior to transplantation after a premedication with glucocorticoids and antihistamines. Based on 3 trials including the ongoing PAES study (NCT05369975) [2, 5], we recommend a strong associated immunosuppressive regimen including steroids, rATG, high dose IVIg, rituximab, tacrolimus and mycophenolic acid. Timing and dosing are particularly important because of the interaction between Imlifidase and immunoglobulins. Our recommendations for these associated treatments are summarized in Figure 4.

### Steroids

Patients will receive decreasing doses of steroids starting on the day of transplantation: 500 mg at day 0, 250 mg from day 1 to day 3, 125 mg on day 4, 20 mg on day 5, then a decrease according to transplant center practice to 5 mg/day at 3 months, with no corticosteroid withdrawal (1A).

### Lymphocyte-Depleting Agents

The two available desensitization studies involving Imlifidase have adopted different lymphocyte depleting strategies: horse ATG (ATGAM) or alemtuzumab. Since horse IgG are not cleaved by Imlifidase, ATGAM is an attractive depleting agent that can be used at day 0 with Imlifidase. However, in France, its use is not approved for kidney transplantation. Alemtuzumab, infused on day 4, is also not available in France for this indication, thus limiting its use. In these studies, it is impossible to compare efficacy between the two regimens since patients receiving ATGAM and those receiving alemtuzumab did not receive identical associated immunosuppression [2, 5].

In more recent publications, both alemtuzumab [49] and ATGAM [50] have been compared to rabbit ATG: rATG has shown repeatedly a better safety and efficacy profile than the two other induction strategies. Imlifidase, on the other hand, cleaves rabbit IgG, and so rATG cannot be infused concomitantly with Imlifidase. However, Imlifidase and rATG interaction has been studied in healthy subjects: 96 h following Imlifidase infusion, cleavage was practically inexistent [1]. We therefore recommend infusion of rATG starting on day 4 at the dose of 1.5 mg/kg/day for a total of 5 days (7.5 mg/kg cumulative dose) (2A). It is important to note that rATG is the lymphocyte-depleting agent used in the ongoing PAES study (NCT05369975).

### IVIg

We also propose high dose IVIg infusion (2 g/kg) over day 4 and 5 in order to reduce the risk of DSA rebound (2C). This approach has been shown to reduce HLA antibodies alone [19] or in association with rituximab [21] in highly sensitized patients awaiting a kidney transplant.

### Rituximab

As for Rituximab, we recommend infusing patients on day 7 post transplantation (375 mg/m^2^ per dose), since it has been shown that it could attenuate the post-transplant DSA rebound [2, 51]. We also propose to infuse rituximab at least 2 weeks before transplantation (2C). However, even a small amount of rituximab in the recipient sera, can render positive a negative crossmatch [52]. In this case it is imperative that the pre-transplant infusion is performed only in centers where the HLA laboratory has the necessary know-how to counter rituximab in crossmatch testing [47, 48]. If this technique is not available, it is recommended not to infuse rituximab before transplantation. However, it is worth noting that the use of rituximab in addition to rATG could increase the risk of leukopenia and infections.

### Standard Maintenance Therapy

Standard maintenance therapy associating tacrolimus and mycophenolate mofetil should be started on day of transplantation with recommended tacrolimus trough levels between 8 and 10 ng/mL and high MPA exposure if tolerated (1A).

### Anti-Infectious Prophylaxis

Because of the hefty immunosuppressive regimen, we strongly encourage patient vaccination for *Pneumococcus pneumoniae* (1A), *Neisseria meningitidis* (serotypes ACWY and B) (1A), Influenza virus (1A) and *SARS COV-2* (1A) prior to inclusion in the program. Once transplanted, patients should undergo CMV and pneumocystis prophylaxis. Further bacterial prophylaxis may also be administered (penicillin). The management of CMV or BK virus infections should be performed according to the most recently published recommendations.

## Monitoring and Follow-Up

**FIGURE 5:**
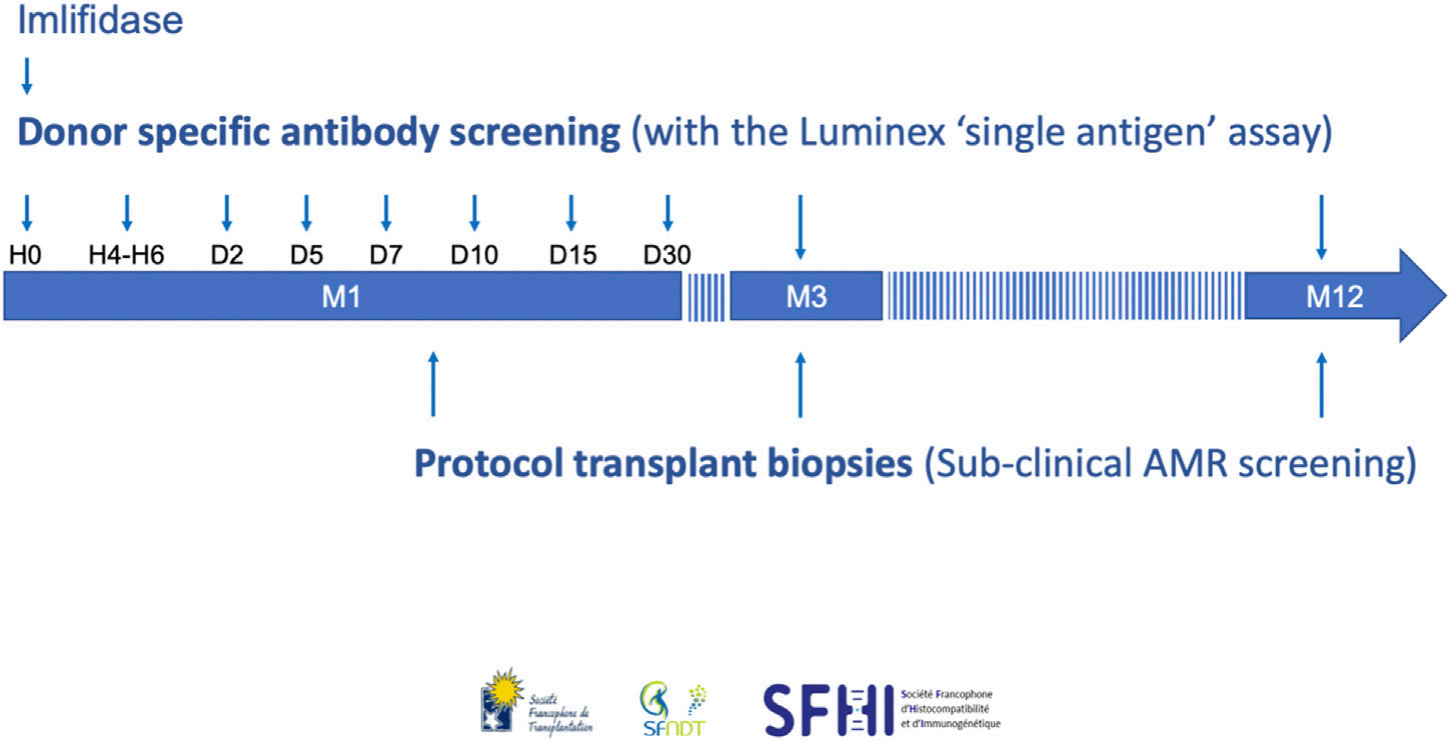
The French consensus guidelines for monitoring and follow-up of patients transplanted with Imlifidase. AMR, antibody-mediated rejection.

Given the DSA rebound and the high rate of AMR, we propose guidelines for the post-transplant management of Imlifidase-treated patients. Our recommendations for serologic and histological monitoring are summarized in Figure 5.

### Donor Specific Antibody

We recommend to test for DSA with the Luminex SAFB technique at the following timepoints:

- Before Imlifidase injection (H0), and 4–6 h (H4-H6) post-injection in order to help interpretation of the pre- and post-Imlifidase XMs (1A).
- At days 2, 5, 7, 10, 15, and month 1, because rebound occurred between day 3 and 14 in the phase II study (1A) [5]. Moreover, a rise in DSA level at week 1 and day 10 was previously associated with AMR [53, 54].
- At months 3 and 12 (1C), because DSA persistence was associated with AMR and a higher risk of chronic AMR in patients transplanted with preformed DSA [55, 56]. At 3 months, persistent DSA was also associated with impaired graft outcome [56, 57].

Moreover, we also recommend that sera be harvested daily during the first week post-transplant and stored, in order to retrospectively and accurately date the onset of a possible rebound or for possible academic purposes (2D).

### Protocol Kidney Biopsies

Systematic surveillance biopsies of the kidney graft are also recommended in all the patients at the following timepoints to detect subclinical rejection: between day 7 and day 10 to capture potential kidney injury at the time of the DSA rebound, and then months 3 and 12 (2C). The incidence of subclinical AMR during the first-year post-transplant in HLA-incompatible kidney transplant recipients has been reported at 80% and more by several teams [58–60]. This incidence is unknown in HLA-incompatible patients treated with Imlifidase. It is then important to clarify this point since subclinical AMR detected at the 1-year screening biopsy leads to a reduced graft survival at 8 years post-transplant (56%) independently of eGFR and proteinuria [61]. Moreover, as subclinical AMR is associated with graft loss, early treatment could be initiated to improve graft outcome [62].

## Conclusion

Imlifidase could be a major breakthrough in kidney transplantation, because this is the first treatment authorized in our field since belatacept more than 10 years ago and could allow transplanting patients so far considered as untransplantable. We urgently need more clinical data coming from clinical trials as well as by unifying efforts across centers and countries, that may enable enhancing the evidence on how to refine the use and implementation of Imlifidase. These French guidelines are partly subjective but are part of this international effort. The experience acquired in the few coming years will help revising and refining them.
